# Progress of the Surgery of the Vermiform Appendix

**Published:** 1903-02-07

**Authors:** 


### Progress of the Surgery of the Vermiform Appendix.

**Appendicitis.**—Miller [1] thinks that in the symptom "dulness" we have an infallible means of differentiating in appendicitis between cases that are operative and non-operative. He believes that when we have a case of appendicitis without the formation of an inflammatory exudate we can afford to wait, but with the occurrence of dulness it is jeopardising the life of the patient to defer operative interference. In an acute attack of appendicitis with dulness, persisting from 24 to 48 hours, and after the bowels have been moved, one should operate, and the chances are pus will be found. In a recurrent attack of appendicitis with dulness, even if the temperature and pulse are normal and the patient is able to be up and about, one should operate, and one will most likely find an indurated appendix with adhesions around the cæcum and appendix. In 41 cases dulness was found in 24, and in the 24 pus was diagnosed by the symptom of dulness in 17. Pain in the region of the appendix does not always mean appendicitis, but localised dulness with the associated symptoms of appendicitis always does. Vignard [2] advocates sub-serous appendicectomy in those instances where the appendix being firmly fixed by old peritoneal adhesions to the parts around, its separation might involve very considerable risk of serious injury to them. The base of the organ is slightly freed and seized with forceps or ligatured, below which it is cut off. The proximal end is treated according to one's usual custom. The cut surface of the distal end is seized about the centre with forceps, and gently drawn upon, while the serous coat is gradually peeled from the appendix until it is completely free, the adhesions remaining undisturbed. Sometimes the base of the appendix is so buried in adhesions that it cannot be reached, only the tip being accessible. In such a case Vignard incised the serous coat circularly just below the tip, upon which he made traction with forceps, and so enucleated the appendix from its bed of adhesions. Joy and Wright [3] give the results of investigations which they have made as to the prognostic value of leucocytosis in cases of appendicitis. After a study of a large number of cases they suggest the following procedure: If the signs, symptoms, and history tend to establish a diagnosis of appendicitis, a leucocyte count is at once made. If the number of the leucocytes is high, say about 16,000 per cubic millimetre, and the symptoms of the case considered by themselves would be called severe, an immediate operation should be strongly recommended. The case is a serious one which may at any time pass beyond the possibility of successful operative relief. If the first count be less than 16,000 (supposing the case has been seen from the commencement of the illness), and subsequent examinations of the blood show no increase, they consider that the case will be a mild one in which the prognosis without operation is good for that particular attack. Bloodgood,[4] in his experience of the leucocyte count in appendicitis comes to the following conclusions:—(1) In a suspected case before the fourth day, a rising leucocytosis indicates operation, *e.g.* if the leucocytosis is 18,000 during the first 48 hours. If less than this, or if the leucocytosis is decreasing hour by hour, an operation is usually unnecessary. (2) After the fourth day, a high leucocytosis indicates a localised abscess. If peritonitis is present there may be but slight leucocytosis, and this drops as the patient becomes more septic. If it is high there is a better chance of recovery. Deaver and Moore [5] strongly condemn the extreme view that the leucocyte count should be made the standard by which operative interference is to be guided in appendicitis, and though recognising its possible value at times, insist that it is very misleading. Kalteyer [6] points out that a leucocyte fall is to be expected when an abscess has become walled off, as the leucocytes have then done their work, and diminish in number to normal. Hewetson [7] believes that the leucocyte count is of considerable value in appendicitis as indicating suppuration. In 23 cases of suppurative appendicitis examined, 80 per cent. gave a leucocytosis above 15,000. In speaking of operation during an acute attack of appendicitis Treves [8] brings forward the following points:— (1) In every case of acute appendicitis of the accepted type there is acute peritonitis. (2) The greater proportion of cases of appendicitis recover spontaneously, and it is probable that the general mortality of the disease—if examples of all grades be included—is not over 5 per cent. (3) Operations carried out during an acute attack are attended by a mortality which is probably over 20 per cent. (4) It must be remembered that relapses may occur after operation carried out during the acute stage. (5) The removal of the appendix during the quiescent period is attended by a mortality of about 1 in 500. (6) The pathology of the disease and its general mortality will not sanction the practice of opening the abdomen in every case of appendicitis as soon as the diagnosis has been established. (7) Immediate operation is demanded, at the earliest possible moment, in all ultra-acute cases. (8) Immediate operation is demanded in every example in which there is reasonable suspicion that suppuration has taken place. (9) In cases outside those mentioned the question of operation may be kept in abeyance for the first few days of the attack, and may usually be left open for decision until the fifth day or after. Riedel [9] says that the old and frequently neglected idea that enteroliths are principally to blame for bad terminations to appendicular inflammation must be held correct. Scarcely one-third of such cases runs a mild course; all the rest are severe. Exceptionally a cure results from the escape of a stone into the cæcum; generally, however, on its departure it leaves behind a stricture or stenosis of the appendix. He considers that acute appendicitis is due to two causes, viz., the presence of eroding enteroliths and of chronic or granulating appendicitis, the latter being an entirely characteristic primary disease. Wyeth [10] thinks that hernia following appendicectomy is less common if the patient is kept in bed for six weeks after operation. Deaver [11] considers that an early operation, preferably in the stage of appendicular colic, is the only rational procedure, and is the only treatment which will reduce the mortality in acute appendicitis to insignificant figures; and that the so-called rest treatment of appendicitis fails to check peritoneal inflammation, and will, in the majority of cases, harm the patient. Heaton,[12] O'Conor [13] and others discuss various points in the pathology and treatment of appendicitis.

[1] Med. Rec., Oct. 18, 1902, p. 613. [2] Med. Chron., March 1902, p. 443. [3] Lancet, April 26, 1902, p. 1199. [4] Quart. Med. Jour., May 1902, p. 240. [5] Ibid., p. 241. [6] Ibid. [7] Ibid. [8] Brit. Med. Jour., June 28, 1902, p. 1593. [9] Annals of Surgery, June 1902, p. 845. [10] Med. Rec., June 7, 1902, p. 893. [11] Ibid., Oct. 4, 1902, p. 554. [12] Birm. Med. Rev., May 1902, p. 265. [13] Lancet, Aug. 16, 1902, p. 427.

